# Microglia depletion fails to abrogate inflammation-induced sickness in mice and rats

**DOI:** 10.1186/s12974-020-01832-2

**Published:** 2020-05-31

**Authors:** Elisabeth G. Vichaya, Sajida Malik, Luba Sominsky, Bianca G. Ford, Sarah J. Spencer, Robert Dantzer

**Affiliations:** 1 Department of Symptom Research, University of Texas MD Anderson Cancer Center, Unit 1055 6565 MD Anderson Boulevard, Houston, TX 77030 USA; 2 Psychology & Neuroscience, Baylor University, Waco, TX 76798-7334 USA; 3 School of Health and Biomedical Sciences, RMIT University, Melbourne, Victoria Australia; 4 ARC Centre of Excellence for Nanoscale Biophotonics, RMIT University, Melbourne, Victoria Australia

**Keywords:** Lipopolysaccharide, Inflammation, Microglia, CSF-1 receptor antagonism, PLX5622, Cx3cr1, Diphtheria toxin, Sickness, Running wheel activity, Mouse, Rat

## Abstract

**Background:**

Production of inflammatory mediators by reactive microglial cells in the brain is generally considered the primary mechanism underlying the development of symptoms of sickness in response to systemic inflammation.

**Methods:**

Depletion of microglia was achieved in C57BL/6 mice by chronic oral administration of PLX5622, a specific antagonist of colony stimulating factor-1 receptor, and in rats by a knock-in model in which the diphtheria toxin receptor was expressed under the control of the endogenous fractalkine receptor (CX3CR1) promoter sequence. After successful microglia depletion, mice and rats were injected with a sickness-inducing dose of lipopolysaccharide according to a 2 (depletion vs. control) × 2 (LPS vs. saline) factorial design. Sickness was measured by body weight loss and decreased locomotor activity in rats and mice, and reduced voluntary wheel running in mice.

**Results:**

Chronic administration of PLX5622 in mice and administration of diphtheria toxin to knock-in rats depleted microglia and peripheral tissue macrophages. However, it did not abrogate the inducible expression of proinflammatory cytokines in the brain in response to LPS and even exacerbated it for some of the cytokines. In accordance with these neuroimmune effects, LPS-induced sickness was not abrogated, rather it was exacerbated when measured by running wheel activity in mice.

**Conclusions:**

These findings reveal that the sickness-inducing effects of acute inflammation can develop independently of microglia activation.

## Introduction

Inflammation induces symptoms of sickness that are characterized by malaise, decreased appetite, fatigue, reduced sociability, increased slow wave sleep, and fever [1]. Experimental studies in rodent models of inflammation confirm that activation of the innate immune system induces behavioral alterations that are reminiscent of sickness and include decreases in locomotor activity, propensity to exercise, and motivation in effort tasks [2]. The mechanisms for these effects involve propagation of inflammation from the periphery to the brain via multiple pathways including afferent nerves, circulating immune mediators interacting with endothelial cells, and macrophages in parts of the brain devoid of a fully functional blood-brain barrier, active transport of immune-derived molecules via the blood-brain barrier and, in some cases, trafficking of peripheral immune cells into the brain [3–6]. This results in the activation of brain microglia and the local production of inflammatory cytokines which, by acting directly or indirectly on neurons, modify brain functions.

The key role of brain microglia in the development of inflammation-induced behavioral alterations has been demonstrated by various approaches mainly aiming at counteracting the production and action of inflammatory cytokines [7] or at normalizing microglial proinflammatory activity and phagocytosis using minocycline [8, 9]. Recently, more targeted approaches have been proposed to eliminate microglia using genetic or pharmacological tools [10]. Based on the observation that the development and survival of microglia critically depends on colony stimulating factor-1 receptor (CSF-1R) signaling [11], CSF-1R antagonists have been successfully developed and are now commonly used to eliminate microglia. Continuous administration of these molecules to mice via their food results in a gradual depletion of Iba-1 and CD68 positive microglia in the brain within a few days of treatment, which persists until cessation of treatment and is then followed by repopulation [10]. As CSF-1R antagonists can have off-target effects, it is useful to compare their effects to those achieved by genetic manipulation of microglia. There are several ways of genetically depleting microglia from knocking out genes that are essential for the survival and development of microglia to administration of immunotoxins such as diphtheria toxin to target the diphtheria toxin receptor genetically inserted in myeloid cells that express the fractalkine receptor CX3CR1 [12]. The objective of the present study was to determine whether ablation of microglia is sufficient to abrogate the behavioral signs of sickness induced by systemic administration of lipopolysaccharide (LPS) to mice and rats. For this purpose, we used the brain penetrant CSF-1R antagonist PLX-5622 [13, 14] in mice and a knock-in rat model in which a diphtheria receptor is expressed under the control of the endogenous *Cx3cr1* promoter sequence [15, 16]. Despite successful depletion of microglia in both models, mice and rats still responded to LPS by behavioral signs of sickness that were concomitant of a neuroinflammatory response.

## Animals and methods

### Animals

Male C57BL/6 J mice (Jackson Labs) were maintained in the MD Anderson animal male facility at 24 °C and 50% humidity. They were provided a control or PLX5622 diet starting at 10 weeks of age. *Cx3cr1-Dtr* rats developed on a Wistar background [15, 16] were maintained at the RMIT University at 22 °C and 40–60% humidity. They were started in experiments between 9–12 weeks of age. All animals were housed on a 12-h light:dark cycle with food and water available ad libitum. All experiments were conducted with approval from their respective animal ethics committee. Rat experiments were conducted in accordance with the Australian Code of Practice for the Care and Use of Animals for Scientific Purposes, with approval from the RMIT University Animal Ethics Committee. Mice experiments were conducted in accordance with the NIH guidelines for care and use of laboratory animals, with approval from the MD Anderson Cancer Center Institutional Animal Care and Use Committee.

### Depletion of microglia and LPS treatment

For the mice experiments, PLX5622 was provided by Plexxikon Inc. (Berkeley, CA). It was formulated in standard AIN-76A rodent chow at a concentration of 1200 mg/kg (Research Diets, New Brunswick, NJ) and provided ad libitum. Control mice were given standard AIN-76A rodent chow. LPS (serotype O127:B8; Sigma-Aldrich, St-Louis, MO) was prepared in a solution of phosphate-buffered saline (PBS) at a concentration of 50 μg/ml and injected intraperitoneally at the dose of 0.5 mg/kg. Control mice received an equivalent volume of PBS.

The knock-in rat model used for depletion of *Cx3cr1* expressing myeloid cells has already been described in detail [15, 16]. *Cx3cr1-Dtr* rats were injected subcutaneously twice with 25 ng/g diphtheria toxin. The injections were separated by an 8-h interval. LPS was injected at the dose of 0.1 mg/kg/ml at 48 h after the first injection of diphtheria toxin, which corresponds to the peak of microglia depletion [15, 16].

### Behavioral testing

Mice were single housed with wireless low-profile running wheels (Med Associates, Fairfax, VT) to measure voluntary wheel running activity, which was quantified as total number of rotations per night (day running is not reported as mice display minimal activity during the day). Running wheels were provided to mice for 10–12 days prior to the initial LPS or PBS treatment to allow the mice to develop stable baseline running behavior. Locomotor activity in a new environment was measured for 5 min after mice were individually placed in an empty rectangular arena (18.4 × 29.2 cm). Activity was recorded by a video camera, and distance traveled was quantified using the Noldus Ethovision XT Software (Noldus Information Technology, Leesberg, VA).

Open-field behavioral testing of rats was performed 2 and 24 h after LPS administration. Each rat was placed into an open-field box of 65 × 65 × 65 cm and filmed for 7 min. The video was analyzed using Ethovision. The arena was divided into two zones: a central zone and an edge zone. The frequency of center entries was assessed as a measure of anxiety, and the distance covered per minute and total distance covered were assessed as measures of locomotor activity. The arena was thoroughly cleaned 70% ethanol between trials and animals.

### Experimental design

The mouse experiment was organized according to a 2 (PLX5622 diet vs. control diet) × 2 (LPS vs. PBS) factorial design with 6 mice per group. The PLX5622 diet or the control diet was administered during the entire duration of the experiment. Mice were group housed with their assigned experimental diet for 12 days before they were single housed and provided with running wheels for the rest of the experiment. LPS or PBS was administered 1 month after the start of experimental diets. Locomotor activity in a new environment was measured 3 h after LPS or PBS treatment, and voluntary wheel running was assessed continuously for 5 days after treatment. One week later, mice were submitted to a cross-over treatment so that mice that had initially received PBS were given LPS and vice versa. They were euthanized for tissue collection 6 h later to assess the effects of PLX5622 on the inflammatory response to LPS.

The rat experiment was organized according to a 2 (*Cx3cr1-Dtr* transgenic rats or wild-type (WT) rats) × 2 (LPS vs. saline) factorial design with 8 rats per group. Rats were given LPS 48 h after diphtheria toxin. Locomotor activity was assessed 2 and 24 h post-LPS. Rats were euthanized for tissue collection immediately following the second locomotor activity assessment.

### Tissue processing

Mice were euthanized by exposure to CO_2_. Livers, and brains were collected after intracardiac perfusion with PBS, snap frozen in liquid nitrogen, and stored at – 80 °C until analyzed. Despite the existence of spatial differences in the mouse brain cytokine response to LPS [17], we decided to study the expression of brain cytokines in the whole brain because the objective of the present study was not to relate neuroinflammatory events possibly occurring in specific brain areas to LPD-induced sickness behavior. RNA was extracted from whole brains using E.Z.N.A. Total RNA Isolation kit (Omega Bio-Tek, Norcross, GA). RNA was reverse transcribed using a High Capacity cDNA Reverse Transcription Kit (Applied Biosystems, Thermo Fisher Scientific, Waltham, MA) and analyzed by real-time PCR in the CFX384 instrument (BioRad) using TaqMan Gene Expression Assays (Applied Biosystems). *Gapdh* was used as a housekeeping gene. Primers are listed in Table 1.

**Table 1:** List of mouse primers

Gene	Accession no.	Foreword sequence	Reverse sequence
*Gapdh*	NM_008084	5′-GTGGAGTCATACTGGAACATGTAG-3′	5′-AATGGTGAAGGTCGGTGTG-3′
*Csf1r*	NM_001037859	5′-TGTATGTCTGTCATGTCTCTGC-3′	5′-AGGTGTAGCTATTGCCTTCG-3′
*Cx3cr1*	NM_009987	5′-TCCCTTCCCATCTGCTCA-3′	5′-CACAATGTCGCCCAAATACAG-3′
*Itgam*	NM_001082960	5′-CCACAGTTCACACTTCTTTCAG-3′	5′-TGTCCAGATTGAAGCCATGA-3′
*Il1b*	NM_008361	5′-GACCTGTTCTTTGAAGTTGACG-3′	5′-CTCTTGTTGATGTGCTGCTG-3′
*Tnf*	NM_013693	5′-AGACCCTCACACTCAGATCA-3′	5′-TCTTTGAGATCCATGCCGTTG-3′
*Il6*	NM_031168	5′-CAAGTGCATCATCGTTGTTCA-3′	5′-GATACCACTCCCAACAGACC-3′
*Il10*	NM_010548	5′-GTCATCGATTTCTCCCCTGTG-3′	5′-ATGGCCTTGTAGACACCTTG-3′
*Oas1a*	NM_145211	5′-GATGAGGATGGCATAGATTCTGG-3′	5′-AGGAGGTGGAGTTTGATGTG-3′

Rats were deeply anesthetized with 150 mg/kg sodium pentobarbitone and were administered intraperitoneally. Livers and brains were collected. Because our previous experiments focused on the hypothalamic neuroendocrine responses to various stimuli in *Cx3cr1-Dtr* rats [15], we decided to continue focusing on this brain area in order to be able to compare the results to those already published. The hypothalamus was dissected from the left hemisphere of the brain over ice. Tissue samples were snap frozen in liquid nitrogen and stored at – 80 °C until analyzed. RNA was extracted from the liver and hypothalamus using QIAzol reagents and RNeasy Mini Kits (Qiagen, Valencia, CA, USA). RNA was reverse transcribed to cDNA using the QuantiTect Reverse Transcription kits (Qiagen) and analyzed by qRT-PCR in the Quantstudi 7 Flex instrument (Applied Biosystems) using Taqman Gene Expression Assays (Applied Biosystems, Mulgrave, VIC, Australia). β-Actin and *Gapdh* were used as housekeeping genes for liver and hypothalamus, respectively. Primers are listed in Table 2.

**Table 2:** List of rat primers

Gene	Accession no.	Taqman assay ID	Product size
*Gapdh*	NM_017008.3	4352338E	63
*Actb*	NM_031144.2	4352340E	91
*Cx3cr1*	NM_133534.1	Rn02134446_s1	124
*IL1b*	NM_031512.2	Rn00580432_m1	121
*Tnf*	NM_012675.3	Rn01525859_g1	92
*Il6*	NM_012589.2	Rn01410330_m1	87
*Il10*	NM_012854.2	Rn01483988_g1	105
*Oas1a*	NM_138913.1	Rn04219673_m1	86

### Data analysis

Data were analyzed by appropriate two-way (PLX vs. LPS or genotype × LPS) or one-way analyses of variance after exclusion of statistical outliers defined by Grubb’s test for rat experiments. Post hoc comparisons of means were performed using Tukey tests or Bonferroni corrections for multiplicity. Data are presented as mean ±standard error of the mean. Statistical significance was defined as *p* < 0.05.

## Results

### Depletion of microglia by PLX5622 does not attenuate LPS-induced neuroinflammation and sickness behavior

#### PLX5622 eliminates microglia in the mouse brain but does not attenuate the brain inflammatory response to LPS

**Fig. 1:**
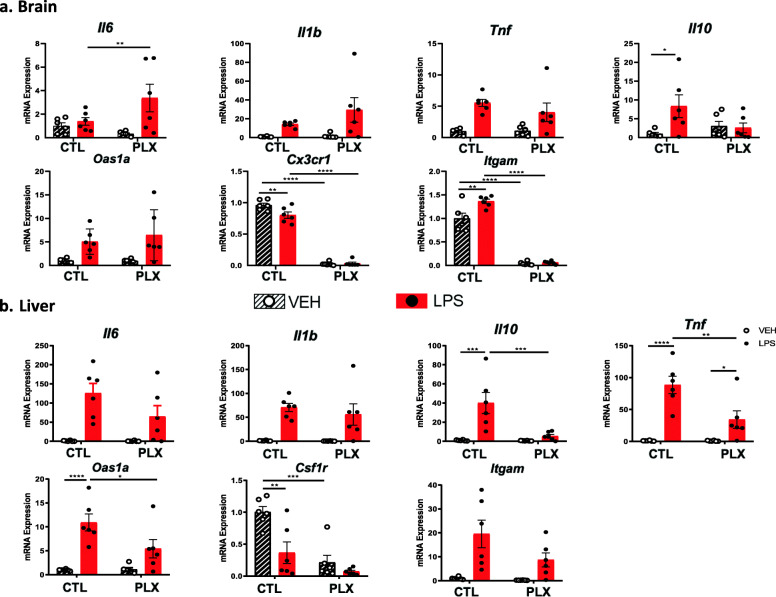
Effects of microglia depletion induced by PLX5622 on the neuroinflammatory response to LPS in the brain and liver. CTL, control diet; PLX, diet supplemented with PLX5622. Mean ± SEM, *n* = 6/group, *p < 0.05, **p < 0.01, ***p < 0.001 (post hoc statistics when significant interaction)

The extent of microglia depletion in mouse brain was quantified by the expression of *Cx3cr1* and *Itgam* mRNA. In accordance with previous reports, PLX5622 abrogated the expression of these microglial markers in the brain (PLX effect *p* < 0.001, Fig. 1a, Table 3). Peripheral macrophages were also depleted by PLX5622 in the liver, as measured by the expression of *Csf1-R* (PLX effect *p* < 0.001, Fig. 1b, Table 3).

**Table 3:** Effects of PLX and LPS on gene expression of markers of microglia/macrophages and proinflammatory cytokines. *F* values (*F*(1, 20)) from 2 (PLX diet vs. control diet) × 2 (LPS vs. control) ANOVA with 6 mice/group

Target molecule	PLX	LPS	PLX) × LPS
Brain *Cx3cr1*	*F*(1, 20) = 684***	*F*(1, 20) = 5.29*	*F*(1, 20) = 6.53*
Brain *Itgam*	*F*(1, 20) = 333***	*F*(1, 20) = 9.61**	*F*(1, 20) = 7.56*
Brain *Il1b*	*F*(1, 20) = 1.43 NS	*F*(1, 20) = 9.70**	*F*(1, 20) = 1.22 NS
Brain *Tnf*	*F*(1, 20) = 0.75 NS	*F*(1, 20) = 21.1***	*F*(1, 20) = 0.95 NS
Brain *Il6*	*F*(1, 20) = 0.30 NS	*F*(1, 20) = 7.56*	*F*(1, 20) = 4.55*
Brain *Il10*	*F*(1, 20) = 1.10 NS	***F*(1, 20) = 3.79+**	*F*(1, 20) = 4.88*
Brain *Oas1a*	*F*(1, 20) = 0.29 NS	*F*(1, 20) = 14.7***	*F*(1, 20) = 0.31 NS
Liver *Csf1r*	*F*(1, 20) = 23.9***	*F*(1, 20) = 12.3**	*F*(1, 20) = 4.95*
Liver *Itgam*	*F*(1, 20) = 3.22 NS	*F*(1, 20) = 17.3***	*F*(1, 20) = 2.42 NS
Liver *Il1b*	*F*(1, 20) = 0.41	*F*(1, 20) = 27.1***	*F*(1, 20) = 0.34
Liver *Tnf*	*F*(1, 20) = 7.96**	*F*(1, 20) = 39.1***	*F*(1, 20) = 7.86*
Liver *Il6*	*F*(1, 20) = 2.49 NS	*F*(1,20) = 23.6***	*F*(1, 20) = 2.44 NS
Liver *Il10*	*F*(1,20) = 9.62**	*F*(1, 20) = 15.2***	*F*(1, 20) = 9.18**
Liver *Oas1a*	*F*(1, 20) = 4.15 NS	*F*(1, 20) = 28.9***	*F*(1, 20) = 4.53*

*NS* non-significant

+*p* < 0.10, *p < 0.05, **p < 0.01, ***p < 0.001

As expected, LPS significantly increased the expression of *Il-1b*, *Tnf*, *IL-6*, and the type I interferon responsive gene *Oas1a* in the brain and liver (LPS effect *p* < 0.05–0.001. Fig. 1a, b). LPS also increased the gene expression of *Il-10* in the brain and liver although it was significant only in the brain (*p* < 0.001). PLX5622 did not alter the brain inflammatory response to LPS with the exception of *Il-6* mRNA which was more highly expressed in the brains of PLX5622-treated mice compared to the brains of control mice in response to LPS (PLX × LPS interaction *p* < 0.05) and *Il-10* mRNA which no longer trended to increase in response to LPS in the brains of PLX5622 mice (PLX × LPS interaction *p* < 0.05, Fig. 1a). In the liver, PLX5622 attenuated the *Tnf*, *Il-10*, and *Oas1a* response to LPS (PLX × LPS interaction *p* < 0.05–0.01) but had no significant effect on the response of other cytokines to LPS (Fig. 1b).

#### PLX5622 does not block the sickness-inducing effects of LPS

**Fig. 2:**
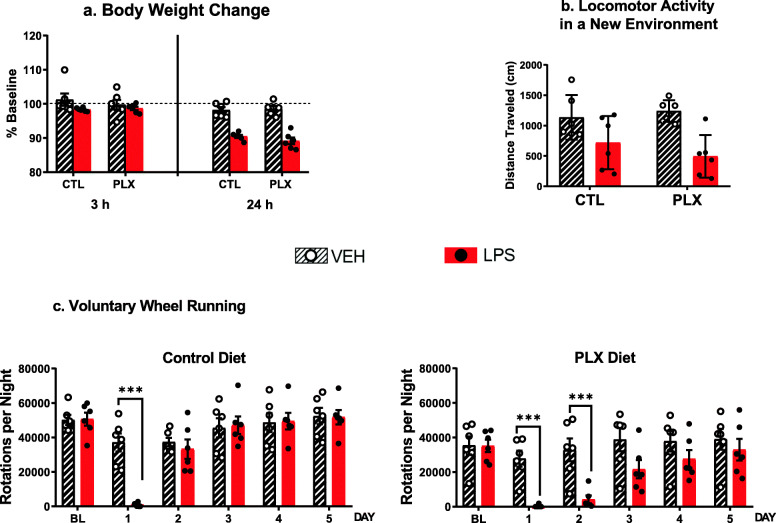
Effects of microglia depletion induced by PLX5622 on the effects of LPS on body weight expressed as percent change from the baseline, locomotor activity in a new environment measured as distance traveled (cm,) and wheel running activity measured by total number of rotations per night at baseline and during 5 days after LPS administration. CTL, control diet; PLX, diet supplemented with PLX5622. Mean ± SEM, *n* = 6/group, *p < 0.05, **p < 0.01, ***p < 0.001

Statistics on the effects of PLX5622 and LPS on body weight and behavior are summarized in Table 4. LPS administration induced body weight loss (Fig. 2a, 24 h vs. baseline, LPS × time *p* < 0.001), and this effect was not modified by PLX5622. LPS decreased locomotor activity in a new environment 3 h after treatment (Fig. 2b, LPS effect *p* < 0.001), and this effect was not modified by PLX. During the week preceding LPS treatment, mice fed the diet supplemented with PLX5622 ran on average 20% less than mice fed the control diet (PLX effect *p* < 0.001) and responded to LPS with a prolonged suppression of voluntary wheel running that lasted 3 days instead of only 1 day for the mice receiving the control diet (Fig. 2c, PLX5622 × LPS × time interaction *p* < 0.001).

**Table 4:** Effects of PLX on body weight, locomotor activity in a new cage, and voluntary wheel running response to LPS. F values from 2 (PLX diet vs. control diet) × time ANOVA for body weight and voluntary wheel running before LPS treatment, from 2 (PLX diet vs. control diet) × 2 (LPS vs. control) ANOVA for locomotor activity in a new cage, and from 2 (PLX diet vs. control diet) × 2 (LPS vs. control) ANOVA with 6 mice/group with time as a repeated factor for body weight loss and voluntary wheel running

	PLX	LPS	PLX × LPS	Time	PLX × time	LPS × time	PLX × LPS × time
Body weight	*F*(1, 22) = 0.200 NS			*F*(6, 132) = 3.85**	*F*(6, 132) = 1.86 NS		
LPS effect on body weight	*F*(1, 20) = 0.342 NS	*F*(1, 20) = 1.14 NS	*F*(1, 20) = 1.31 NS	*F*(2, 40) = 65.2***	*F*(2, 40) = 1.57 NS	*F*(2, 40) = 31.0***	*F*(2, 40) = 1.57 NS
LPS effect on activity new cage	*F*(1, 20) = 0.186 NS	*F*(1, 20) = 16.8***	*F*(1, 20) = 1.36 NS				
Pre-LPS wheel running	*F*(1, 22) = 18.4***			*F*(6, 132) = 44.3***	*F*(6, 132) = 0.803 NS		
LPS effect on wheel running	*F*(1, 20) = 275***	*F*(1, 20) = 7.45*	*F*(1, 20) = 1.67 NS		*F*(5, 100) = 4.24**	*F*(5, 100) = 25.8***	*F*(5, 100) = 7.90***

*NS* non-significant

*p < 0.05, **p < 0.01, ***p < 0.001

### Depletion of microglia by diphtheria toxin in knock-in rats does not attenuate LPS-induced neuroinflammation and sickness behavior

#### Administration of diphtheria toxin to *Cx3cr1-Dtr* rats eliminates microglia but does not abrogate LPS-induced neuroinflammation

**Fig. 3:**
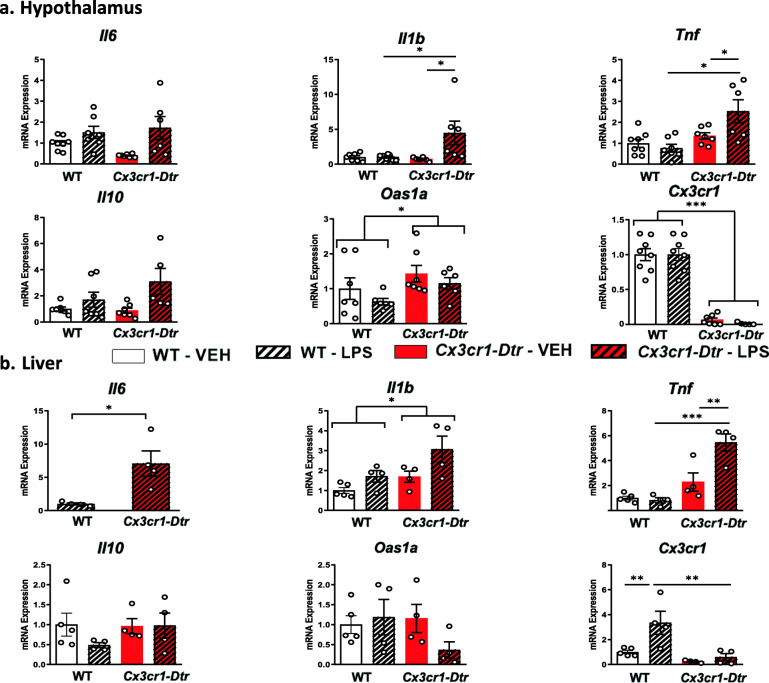
Effects of microglia depletion induced by administration of diphtheria toxin to *Cx3cr1-Dtr* transgenic rats on the neuroinflammatory response to LPS in the hypothalamus and liver. LPS (0.5 mg/kg) was administered 48 h after diphteria toxin was given to ablate microglia, and tissue samples were collected 24 h later. Mean ± SEM, *n* = 4–8/group, *p < 0.05, **p < 0.01, ***p < 0/001

The extent of microglia depletion was quantified by the expression of *Cx3cr1* mRNA in the rat hypothalamus. As expected, administration of diphtheria toxin abrogated the expression of this microglial marker in the hypothalamus at 72 h after DT (DT effect *p* < 0.001, Fig. 3a, Table 5). Peripheral macrophages were also depleted in the liver of rats injected with diphtheria toxin, as measured by the gene expression of *Cx3cr1* at the same time point (DT effect *p* < 0.01 Fig. Fig. 3b, Table 5). Of note, microglia depletion was associated with an increased expression of the interferon-dependent gene *Oas1a* in the hypothalamus (DT effect *p* < 0.05) but not in the liver.

**Table 5:** Effects of microglial depletion by diphtheria toxin on the effects of LPS on gene expression of markers of microglia/monocytes and proinflammatory cytokines in the brain (hypothalamus) and liver of *Cx3cr1-Dtr* rats. *F* values from 2 (diphtheria toxin (DT) vs. control) × 2 (LPS vs. control) ANOVA with 4-8 rats/group. Liver expression of IL-6 was undetectable in saline-treated wild-type and *Cx3cr1-Dtr* rats. LPS-treated groups were therefore compared by a Student unpaired *t* test

Target molecule	DT	LPS	DT × LPS
Brain *Cx3cr1*	*F*(1, 23) = 124***	*F*(1, 23) = 1.14 NS	*F*(1,23) = 0.16 NS
Brain *Il1b*	*F*(1, 22) = 4.05 NS	*F*(1, 22) = 5.63*	*F*(1, 22) = 5.47*
Brain *Tnf*	F(1, 24) = 13.8**	*F*(1, 24) = 2.78 NS	*F*(1, 24) = 6.17*
Brain *Il6*	F(1, 24) = 0.46 NS	*F*(1, 24) = 10.64**	*F*(1, 24) = 2.10 NS
Brain *Il10*	F(1, 22) = 1.55 NS	*F*(1, 22) = 8.40**	*F*(1, 22) = 2.27 NS
Brain *Oas1a*	*F*(1, 22) = 4.31*	*F*(1, 22) = 1.20 NS	*F*(1, 22) = 0.4 NS
Liver *Cx3cr1*	*F*(1, 13) = 14.2**	*F*(1, 13) = 8.42*	***F*(1, 13) = 4.58+**
Liver *Il1b*	*F*(1, 13) = 7.45*	*F*(1, 13) = 7.78*	*F*(1, 13) = 0.79 NS
Liver *Tnf*	*F*(1, 13) = 9.35**	*F*(1, 13) = 36.4***	*F*(1, 13) = 11.7**
Liver *Il6*			*t*(6) = 3.19*
Liver *Il10*	*F*(1, 13) = 0.82 NS	*F*(1, 13) = 1.02 NS	*F*(1, 13) = 1.12 NS
Liver *Oas1a*	*F*(1, 13) = 1.15 NS	*F*(1, 13) = 0.92 NS	*F*(1, 13) = 2.43 NS

*NS* non-significant

+*p* < 0.10, *p < 0.05, **p < 0.01, ***p < 0.001

At 24 h after LPS (72 h after DT), the *Il1b* and *Tnf* mRNA levels were indistinguishable in control rats from those treated with saline, but these levels were significantly elevated in the *Cx3cr1-Dtr* rats (DT × LPS interaction *p* < 0.05, Fig. 3a, Table 5). This indicates an exacerbated neuroinflammatory response to LPS or a delayed recovery. The same pattern was observed in the liver for *Il6* and *Tnf* at this same time 24 h after LPS (DT × LPS interaction *p* < 0.05, Fig. 3b, Table 5).

#### Administration of diphtheria toxin to *Cx3cr1-Dtr* rats does not block the sickness inducing effects of LPS

**Fig. 4:**
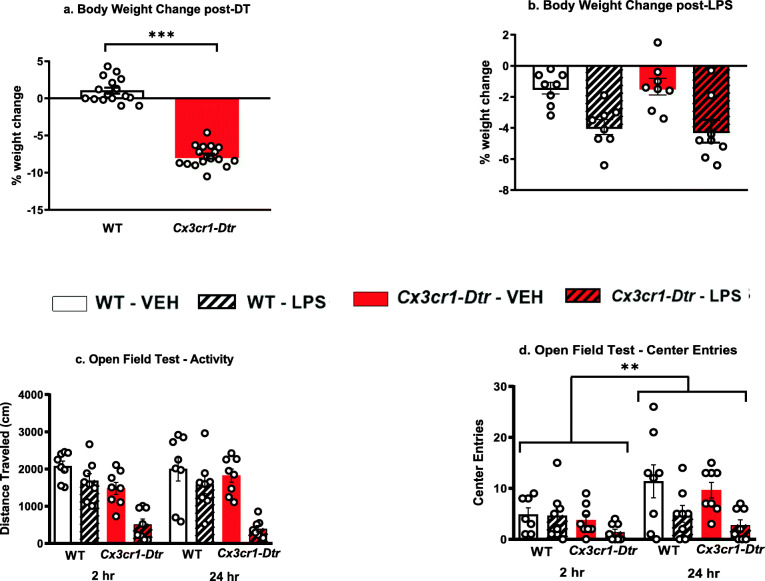
Effects of diphtheria toxin on **a** body weight measured 48 h after in *Cx3cr1-DTr* transgenic rats compared to wild-type (WT) rats, **b** on LPS-induced body weight changes measured 24 h post-LPS, and **c-d** on locomotor activity measured by distance traveled and center entries in an open-field test carried out 2 h and 24 h post-LPS. Means ± SEM, *n* = 8/group except for (**a**), **p < 0.01, ***p < 0.001

As described previously, administration of diphtheria toxin caused significant body weight loss by 48 h (DT effect *p* < 0.001, Fig. 4a, Table 6). LPS caused further weight loss 24 h after treatment (LPS effect *p* < 0.001, Fig. 4b) but the degree of loss did not differ between control and diphtheria toxin-treated rats. LPS reduced total locomotor activity in the open-field at 2 and 24 h after treatment only in those rats which had received diphtheria toxin (DT × LPS interaction *p* < 0.05, Fig. 4c, Table 6). There was a significant LPS treatment by time interaction for the number of center entries in the open field (LPS × time interaction *p* < 0.05, Fig. 4d, Table 6), with an increase in center entries at 24 h compared to 2 h for the saline-treated group but not the LPS-treated group. However, there were no differences between the controls and diphtheria toxin-treated rats on this measure, which can be interpreted as indicating that microglia/monocyte ablation did not affect this form of anxiety-like behavior.

**Table 6:** Effects of microglial depletion by diphtheria toxin in *Cx3cr1-Dtr* rats on the effects of LPS on body weight and activity and center entries in the open-field test. Body weight differences post-DT are assessed by a Student *t* test, comparing all wt with all *Cx3cr1-Dtr* rats (16–17 rats per group). *F* values from 2 (diphtheria toxin vs. control) × 2 (LPS vs. control) and time as the repeated measures ANOVA with 7–9 rats/group

	DT	LPS	DT × LPS	Time	DT × time	LPS × time	DT × LPS × time
Body weight	*t*(31) = 16.6***						
LPS effect on body weight	*F*(1, 28) = 0.03 NS	*F*(1, 28) = 24.3***	*F*(1, 28) = 0.12 NS				
LPS effect on activity in the open field	*F*(1, 28) = 20.7***	*F*(1, 28) = 21.6***	*F*(1, 28) = 5.21*	*F*(1, 28) = 0.01 NS	*F*(1, 28) = 1.19 NS	*F*(1, 28) = 1.84 NS	*F(*1, 28) = 1.44 NS
LPS effect on center entries in the open field	*F*(1, 26) = 3.55 NS	*F*(1, 26) = 12.2**	*F*(1, 26) = 0.08 NS	*F*(1, 26) = 8.80**	*F*(1, 26) = 0.01 NS	*F*(1, 26) = 4.68*	*F*(1, 26) = 0.24 NS

*NS* non-significant

*p < 0.05, **p < 0.01, ***p < 0.001

## Discussion

The present results show that microglia/macrophage depletion either by PLX5622 in mice or by immunotoxin in transgenic rats failed to abrogate the peripheral and central inflammatory response to LPS. Therefore, it was not surprising that this treatment was unable to prevent the signs of sickness that developed in response to LPS. These unexpected findings indicate that the sickness-inducing effects of systemic inflammation can occur independently from microglial activation.

As already reported in previous studies on CSF-1R antagonism [10, 11, 14], administration of the CSF-1R antagonist PLX5622 for 4 weeks resulted in the near complete elimination of microglia in the brain and a significant depletion of macrophages in the spleen and liver. An alternative to the use of CSF-1R antagonism to deplete microglia is the diphtheria toxin receptor-mediated cell knockout technique. This technique is widely used to remove specific cell types in rodents engineered to express the diphtheria toxin receptor on the surface of a specific cell type [18]. Several variants of this technique have already been used to efficiently deplete microglia in mice [12, 19, 20] and in rats [15, 16] by coupling the diphtheria toxin receptor to the promoter of the gene coding for the microglia/monocyte-specific marker CX3CR1. Diphtheria toxin itself is generally well tolerated when administered to wild-type mice [21]. In the absence of diphtheria toxin, *Cx3cr1-Dtr* transgenic rats do not show any abnormalities [15, 16]. Similar to mouse models utilizing conditional diphtheria toxin receptor expression approach [12, 22, 23], administration of diphtheria toxin in *Cx3cr1-Dtr* rats depleted microglia by 48 h in various brain regions including the hypothalamus, with repopulation occurring by 7 days [15, 16]. Although microglia depletion was associated with anorexia and weight loss, this was not due to sickness as there was no changes in locomotor activity in an open-field and in two tests of anxiety, the elevated plus maze, and the light-dark box [15]. There was also no indication of nausea as measured by ingestion of kaolin. In addition, microglia depletion by diphtheria toxin was not associated with any evidence of impairment in learning and memory as measured by short-term memory in a novel object and place recognition tasks [16]. Further studies indicate that the anorexia induced by administration of diphtheria toxin to *Cx3cr1-Dtr* rats is actually due to disruption of the gustatory circuitry a the level of the paraventricular nucleus of the hypothalamus [15], indicating the complex role microglia play in brain functions additional to their traditional role in regulating neuroinflammation [24].

We anticipated that the elimination of microglia by PLX5622 in mice and by diphtheria toxin in *Cx3cr1-Dtr* rats would attenuate neuroinflammation induced by LPS and its behavioral consequences. In accordance with this prediction, there are already several publications showing that depletion of microglia by PLX5622 protects from neuroinflammation [25–28] and prevents behavioral alterations in response to cranial irradiation [28], repeated social defeat [29], partial sciatic nerve ligatio n[30], and experimental autoimmune encephalomyelitis [27]. In addition, antibody-mediated neutralization of peripheral macrophage CSF-1R was reported to block the development of sickness behavior measured by reduced locomotor activity and body weight loss in response to CD40 activation, a model of autoimmune disease [31].

It is currently unclear why the elimination of microglia/macrophages by CSF-1R antagonism or by diphtheria toxin in the *Cx3cr1-Dtr* rat model failed to abrogate the inflammatory and behavioral response to LPS. At the periphery, this could be due to the fact that both interventions specifically depleted tissue macrophages but did not affect pro-inflammatory monocytes recruited from the bone marrow, dendritic cells, or neutrophils which can all contribute to the peripheral inflammatory response [32]. However, this cannot explain why the brain response to LPS was not only not fully abrogated in both models of microglia depletion but actually enhanced in *Cx3cr1-Dtr* rats. We note that LPS-treated *Cx3cr1-Dtr* rats displayed a rapid (2 h) reduction in the open-field behavior that persisted until 24 h, suggesting sickness behaviors that are, if anything, exacerbated in the absence of microglia. Cytokine responses were also elevated at that time point. We have previously seen no effect of microglia ablation per se on behavioral indices of sickness including open-field, elevated plus maze, light/dark box, or ingestion of kaolin clay [15]. However, it is possible that while microglia ablation does not itself lead to an inflammatory response, the brain is primed to hyper respond to further challenge. Indeed, we have also shown astrocytes are hyper-phagocytic of microbeads in brain slice preparations in the absence of microglia [16].

In the first study to show that CSF1 receptor antagonism eliminates microglia in a reversible way, mice were treated with a low dose of LPS (0.25 mg/kg) after only 7 days of the CSF-1R antagonist PLX3397, and brains were collected 6 h after LPS without intracardiac perfusion to eliminate residual blood [11]. While this study showed that PLX3397 attenuated IL-1β and reversed TNF mRNA expression in response to LPS, it had only limited effects on other inflammatory markers, with no effect on IL-6 mRNA expression in response to LPS. In addition, a number of studies show that microglial depletion is not always neuroprotective. In mice infected with prions, administration of PLX5622 accelerated disease progression [33]. In the same manner, PLX5622 increased viral load and enhanced mortality in a number of murine models of viral infection [22, 23, 34]. A similar protective role of microglia was also apparent in the progression of neurodegeneration in APP-PS1 transgenic mice [35], the extent of excitotoxic injury in a model of brain injury induced by cerebral ischemia [36], and the dopaminergic neurotoxicity of 1-methyl-4-phenyl-1,2,3,6-tetrahydropyrine (MPTP) [37].

One possibility for the conserved production of cytokines despite microglia depletion is the well-known existence of genetically defined subsets of microglia in the brain [38–40] with differential sensitivity to genetic or pharmacological depletion. The techniques used to induce microglia depletion leave intact a very small percentage of microglia in the brain, less than 1% in response to CSF-1R antagonism [41]. This resistant subset of microglia has been identified as having distinct self-renewal capacity following depletion and repopulation [41]. However, its ability to produce cytokines in response to neuroinflammation has not been examined, and it is difficult to imagine that it is sufficient to induce a similar and even higher inflammatory response to LPS than the whole brain microglia population.

Another possibility is the compensation of microglia functions by other brain cell types including astrocytes, oligodendrocytes, pericytes, and endothelial cells. In particular, endothelial cells are well known to play an important role in the transmission of the peripheral inflammatory message to the brain as they respond to inflammatory cytokines such as IL-1β by production of inflammatory mediators [42, 43]. In the absence of investigation of LPS-induced cytokine production at the cellular level in the present study, we cannot determine which exact brain cell types are mediating the exacerbated brain response to LPS after microglia depletion. We have already reported that in *Cx3cr1-Dtr* rats, the density of astrocytes and their phagocytic activity are increased [16]. Other studies point to a likely role of astrocytes. In the study on MPTP [37], flow cytometry analysis of chemokines and proinflammatory cytokines in astrocytes from the substantia nigra and striatum revealed that PLX5622 significantly increased the IL-6 and TNF response to MPTP. These findings can be interpreted to suggest that microglia cells downregulate the astrocytic response to inflammatory insults. There is already evidence that astrocytes from mice treated chronically with the CSF-1R antagonist PLX3397 to deplete microglia still respond to LPS in vivo by developing a reactive A1 phenotype [44]. This is probably facilitated by the lack of IL-10 from microglial origin as this anti-inflammatory cytokine normally lowers the proinflammatory profile of LPS-activated astrocytes [45]. Activation of an astrocyte-dependent type 1 interferon response was also proposed to account for the gray matter neurodegeneration that was observed at a late stage in a model of diphtheria toxin-induced microglia depletion in a *Cx3cr1-CreER* mouse system [46]. The possibility that reactive A1 astrocytes induced by LPS take over in the absence of microglia is consistent with the observation that in our study brain IL-6, a cytokine mainly produced by astrocytes during neuroinflammation [47], was the only cytokine of which the gene expression in response to LPS was enhanced by PLX5622. The increased expression of the interferon-dependent gene *Oas1a* in the hypothalamus of diphtheria toxin-treated transgenic rats follows the same direction of change.

Another mechanism for the lack of attenuation of neuroinflammation by microglia depletion could be an enhanced trafficking of immune cells into the brain of microglia-depleted mice. However, this is unlikely to account for the present results as it has been shown that PLX3397 treatment does not compromise the integrity of the blood-brain barrier, based on blue Evans coloration exclusion [11]. In addition, in situations in which there was evidence of increased infiltration of lymphocytes in the brain of microglia-depleted mice, genetic elimination of lymphocytes did not modify the increased sensitivity of microglia-depleted mice to neurodegeneration [37]. The possible existence of a compromised blood-brain barrier has not yet been examined in the diphtheria toxin-induced transgenic model.

There has been no previous attempt to assess the effect of microglial depletion on the ability of rodents to engage in strenuous exercise, as measured by voluntary wheel running activity or by treadmill running. Our results show that PLX5622 decreased the amount of voluntary wheel running at baseline by about 20%. It is possible to interpret this finding in the context of what is already known concerning the involvement of microglia in the beneficial effects of physical exercise. In particular, microglial activation within the neurogenic niche has been shown to mediate the beneficial effects of running wheel activity on hippocampal neurogenesis in the adult or aged mouse brain [48, 49]. In addition, wheel running has been reported to induce microglia proliferation in the adult murine cortex, which could play a role in the positive effects of physical exercise on neurological health [50, 51]. Our observation of a significant decrease in voluntary wheel running activity in microglia-depleted mice is consistent with this hypothesis.

Besides the lack of investigation of the cytokine response at the cellular level to determinate which brain cell types continue to respond to LPS after microglia depletion, our study has a few other limitations. One limitation is the lack of a time course analysis of the cytokine response to LPS. In the mouse experiment, we examined the cytokine response at only 6 h post-LPS as the main objective which was to assess the effect of PLX5622 on LPS-induced expression of peripheral and brain cytokines and not to explain the delayed recovery of wheel running behavior in PLX5622-treated mice. In the rat experiment, we examined the cytokine response at only 24 h post-LPS as we already know that at this time, there is normally no more cytokine expressed in the hypothalamus [52, 53]. The fact we still observed inflammatory cytokine expression in the brain of transgenic rats in response to LPS at this time despite microglia ablation while control rats showed no change can therefore be interpreted safely as evidence of a delayed recovery of the cytokine response to LPS.

Another limitation is the absence of investigation of possible sex differences. We were unable to assess possible sex differences in the extent of microglia depletion induced by CSF-1R antagonism in mice or by immunotoxin in transgenic rats and in the effects of microglia depletion on the inflammatory and behavioral response to LPS as all the experiments were carried out in males. However, experiments carried out with PLX5622 and PLX3397 revealed no sex differences in the extent of microglia depletion induced by either of these treatments [33, 35, 54–57]. In the same manner, female and male *Cx3cr1-Dtr* rats were found to respond identically to diphtheria toxin administration in terms of microglia depletion and body weight loss [15]. This does not eliminate the possibility of an interaction between microglial depletion and the effect of the intervention, LPS in this case, as such an interaction has been described for the effects of microglial depletion by PLX3397 in rats fed a high fat diet. Microglia depletion protected only male but not female mice from the deleterious effects of a high fat diet on executive function [58].

## Conclusion

In conclusion, the results of the present study carried out in two different models of microglia elimination and two different animal species cast doubt on an exclusive role of microglia activation in the sickness inducing effects of systemic inflammation.

## Data Availability

The datasets collected and analyzed during the current study are available from the corresponding author on reasonable request.

## Abbreviations

Cd11b: Cluster of differentiation 11b
CSF-1: Colony stimulating factor 1
CSF-1R: Colony stimulating factor 1 receptor
CX3CR1: CX3C chemokine receptor 1
Dtr: Diphtheria toxin receptor
E.Z.N.A.: Registered commercial name
IL-1β: Interleukin-1beta
IL-6: Interleukin-6
IL-10: Interleukin-10
LPS: Lipopolysaccharide
mRNA: Messenger ribonucleic acid
Oas1a: 2′-5′-oligoadenylate synthase 1A
PBS: Phosphate-buffered saline
PCR: Polymerase chain reaction
TNF: Tumor necrosis factor-alpha
WT: Wild-type
